# An Updated Meta-Analysis of Endothelial Nitric Oxide Synthase Gene: Three Well-Characterized Polymorphisms with Hypertension

**DOI:** 10.1371/journal.pone.0024266

**Published:** 2011-09-02

**Authors:** Wenquan Niu, Yue Qi

**Affiliations:** 1 State Key Laboratory of Medical Genomics at Ruijin Hospital, Shanghai Jiao Tong University School of Medicine, Shanghai, China; 2 Laboratory of Vascular Biology, Institute of Health Sciences, Shanghai Institutes for Biological Sciences, Chinese Academy of Sciences, Shanghai, China; 3 Shanghai Key Laboratory of Vascular Biology, Shanghai Institute of Hypertension, Shanghai Jiao Tong University School of Medicine, Shanghai, China; 4 Department of Epidemiology, Beijing An Zhen Hospital, Beijing Institute of Heart, Lung and Blood Vessel Diseases, Capital Medical University, Beijing, China; Leiden University Medical Center, The Netherlands

## Abstract

**Background:**

Numerous individually underpowered association studies have been conducted on endothelial nitric oxide synthase (*eNOS*) genetic variants across different ethnic populations, however, the results are often irreproducible. We therefore aimed to meta-analyze three *eNOS* widely-evaluated polymorphisms, G894T (rs1799983) in exon 7, 4b/a in intron 4, and T−786C (rs2070744) in promoter region, in association with hypertension from both English and Chinese publications, while addressing between-study heterogeneity and publication bias.

**Methods:**

Data were analyzed using Stata software (version 11.0), and random-effects model was applied irrespective of between-study heterogeneity, which was evaluated by subgroup and meta-regression analyses. Publication bias was weighed using the Egger's test and funnel plot.

**Results:**

There were total 19284/26003 cases/controls for G894T, and 6890/6858 for 4b/a, and 5346/6392 for T−786C polymorphism. Overall comparison of allele 894T with 894G in all study populations yielded a 16% increased risk for hypertension (odds ratio [OR] = 1.16; 95% confidence interval [95% CI]: 1.07–1.27; P = 0.001), and particularly a 32% increased risk (95% CI: 1.16–1.52; P<0.0005) in Asians and a 40% increased risk (95% CI: 1.19–1.65; P<0.0005) in Chinese. Further subgroup analyses suggested that published languages accounted for the heterogeneity for G894T polymorphism. The overall OR of allele 4a versus 4b was 1.29 (95% CI: 1.13–1.46; P<0.0005) in all study populations, and this estimate was potentiated in Asians (OR = 1.42; 95% CI: 1.16–1.72; P<0.0005). For T−786C, ethnicity-stratified analyses suggested a significantly increased risk for −786C allele (OR = 1.25; 95% CI: 1.06–1.47; P = 0.007) and −786CC genotype (OR = 1.69; 95% CI: 1.20–2.38; P = 0.003) in Whites. As an aside, the aforementioned risk estimates reached significance after Bonferroni correction. Finally, meta-regression analysis on other study-level covariates failed to provide any significance for all polymorphisms.

**Conclusion:**

We, via a comprehensive meta-analysis, ascertained the role of *eNOS* G894T and 4b/a polymorphisms on hypertension in Asians, and T−786C polymorphism in Whites.

## Introduction

Hereditary factors contributed directly to the occurrence of hypertension, as evidenced by family studies showing that premature onset of hypertension among first-degree relatives yielded a remarkable high risk of 3.8 times to develop this disorder [1]. However, it remains unclear how many genes or which genetic determinants might constitute such hereditary background [2]. The gene encoding endothelial nitric oxide synthase (protein: eNOS; gene: *eNOS*) is regarded as one of the potentially logical candidate for hypertension, since its enhanced production or enzyme bioavailability can lead to the constitutive release of nitric oxide in endothelial cells, which exerts vasoprotective effects in blood pressure (BP) regulation [3].

The *eNOS* spans 21 kb with 26 exons on chromosome 7q35-36. The biological candidacy of *eNOS* in hypertension has been well-defined. For example, knockout mice deficient in *eNOS* developed hypertension as adults [4], and contrastingly induction of *eNOS* cDNA in mice reduced BP [5]. Since the genomic sequence of *eNOS* is highly polymorphic, it is of added interest to confirm which polymorphism(s) at *eNOS* might have functional potentials to affect the final bioavailability of eNOS, and thus the development of hypertension. A large panel of individually underpowered studies have been conducted on *eNOS* polymorphisms across different ethnic populations, yet with irreproducible and inconclusive results. To derive a more precise estimation, we therefore meta-analyzed three *eNOS* widely-evaluated polymorphisms, G894T (rs1799983) in exon 7, 4b/a (an insertion-deletion with 4a denoting four tandem 27-bp repeats and 4b five repeats) in intron 4, T−786C (rs2070744) in promoter region, in association with hypertension from the English and Chinese-published literature, while addressing between-study heterogeneity and publication bias.

## Methods

### Literature Search

Publications were identified from two international searching engines, *viz*. PubMed and Excerpta Medica Database (EMBASE), as well as two Chinese searching engines, *viz*. Wanfang database (http://www.wanfangdata.com.cn) and China Biological Medicine (CBM) (http://sinomed.imicams.ac.cn/index.jsp) as of Dec. 31, 2010. As a prerequisite, we restricted the search spectrum to articles written in English or Chinese language, and studies in human subjects. Boolean combinations of the key subjects (hypertension OR blood pressure) AND (endothelial nitric oxide synthase OR nitric oxide synthase 3 OR eNOS OR NOS3 OR ECNOS) AND (allele OR genotype OR polymorphism OR variant OR variation) were used for identification. The full text of the retrieved articles was scrutinized to inspect whether data on the topic of interest were included. Also, reference lists of the retrieved articles and systematic reviews were checked to determine whether citations of articles that were not initially identified. If more than one geographical or ethnic populations were included in one report, each population was treated separately.

### Inclusion/Exclusion Criteria

Identified studies satisfied the following criteria: (i) evaluation of *eNOS* G894T or 4b/a or T−786C polymorphism in association with hypertension; (ii) case-control or nested case-control or cross-sectional study; (iii) genotype/allele counts of G894T, 4b/a and T−786C polymorphisms between cases and controls for estimating odds ratio (OR) and 95% confidence interval (95% CI).

Hypertension was defined as systolic BP equal to or above 140 mmHg or diastolic BP equal to or above 90 mmHg or treatment with antihypertensive medication. Moreover, gestational hypertension studies were also included. However, studies evaluating secondary hypertension or other types of monogenic hypertension were excluded. If two or more studies shared the whole or part of study populations, the one with larger sample size was recruited.

### Extracted Information

Two authors (W. Niu and Y. Qi) independently drew the following information from all qualified studies: first author's last name, publication date, population ethnicity, diagnosis criteria for BP, study design, baseline characteristics of the study population, and the genotype distribution in patients and controls. Any encountered discrepancies were adjudicated by a discussion until a consensus was reached.

For consistency, continuous variables expressed as mean ± standard error (SE) were converted to mean ± standard deviation (SD). Moreover, the units of measures used in this study are transformed into the standard measurement units.

### Statistical Analysis

Overall allelic/genotypic associations of *eNOS* G894T, 4b/a and T−786C polymorphisms with hypertension were calculated using the Stata software version 11.0 for Windows. In this meta-analysis, we implemented the random-effects model using the method of DerSimonian & Laird, instead of fixed-effects model, to bring the individual effect-size estimates together, and the estimate of heterogeneity was taken from the Mantel-Haenszel model [6].

Heterogeneity was assessed by the *I* ^2^ statistic, which was documented for the percentage of the observed between-study variability due to heterogeneity rather than chance with the ranges of 0 to 100% *[I* ^2^ = 0–25%, no heterogeneity; *I* ^2^ = 25–50%, moderate heterogeneity; *I* ^2^ = 50–75%, large heterogeneity; *I* ^2^ = 75–100%, extreme heterogeneity] [7].

In addition, to look at more narrowly drawn subsets of the studies such as different study designs, separate analyses were undertaken in a sensitivity manner. Furthermore, to estimate the extent to which one or more covariates explain heterogeneity, meta-regression, as an extension to random-effects meta-analysis, was employed.

Finally, publication bias was assessed using the Egger regression asymmetry test. The Egger's test detects funnel plot asymmetry by determining whether the intercept deviates significantly from zero in a regression of the standardized effect estimates against their precision.

A naïve probability of less than 0.05 was judged significant, and Bonferroni correction was used to control for the multiple testing in view of three polymorphisms under investigation (significance was set at 0.05/3). Because the inconsistency index *I* ^2^ test and Egger's test are recognized to have poor power, it is recommended that these statistics were statistically significant if the probability was less than 0.1 [7].

## Results

### Description of Available Studies

**Figure 1:**
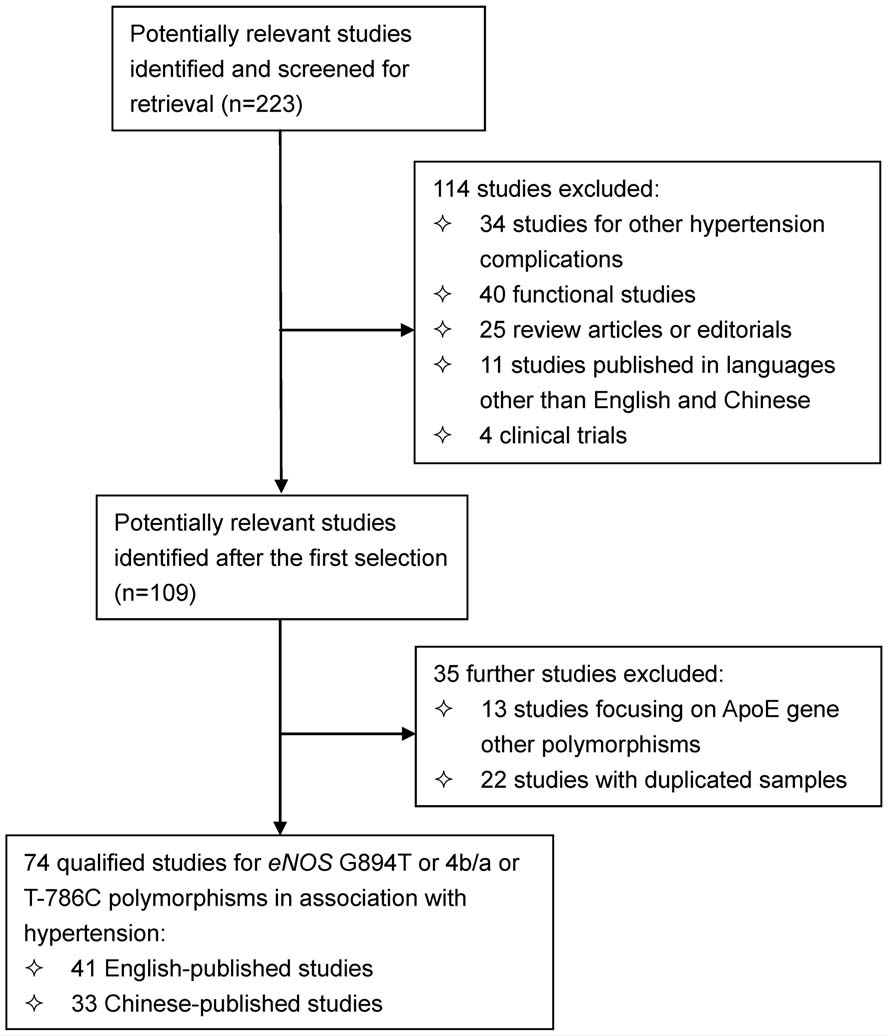
Flow diagram of search strategy and study selection.

According to our search strategies, total 115 written-in-English (WIE) studies and 108 written-in-Chinese (WIC) studies were initially identified. Further application of our inclusion/exclusion criteria left 41 WIE (43 populations) [8]–[48] and 33 WIC (34 populations) (See Table S1: Chinese references 1–33) studies. Thereof, 32 WIE and 23 WIC studies were qualified for G894T polymorphism, 21 and 12 for 4b/a polymorphism, and 12 and 4 for T−786C polymorphism. As for the sample size, there were 19284 (WIE/WIC: 15498/3783) cases and 26003 (22444/3559) controls for G894T polymorphism, and 6890 (4763/2127) cases and 6858 (4816/2042) controls for 4b/a polymorphism, and 5346 (4693/653) cases and 6392 (5956/436) controls for T−786C polymorphism. The baseline characteristics of all qualified studies and a diagram schematizing the selection process of identified studies are presented in Table S1 and Figure 1, respectively.

### G894T and Hypertension

**Figure 2:**
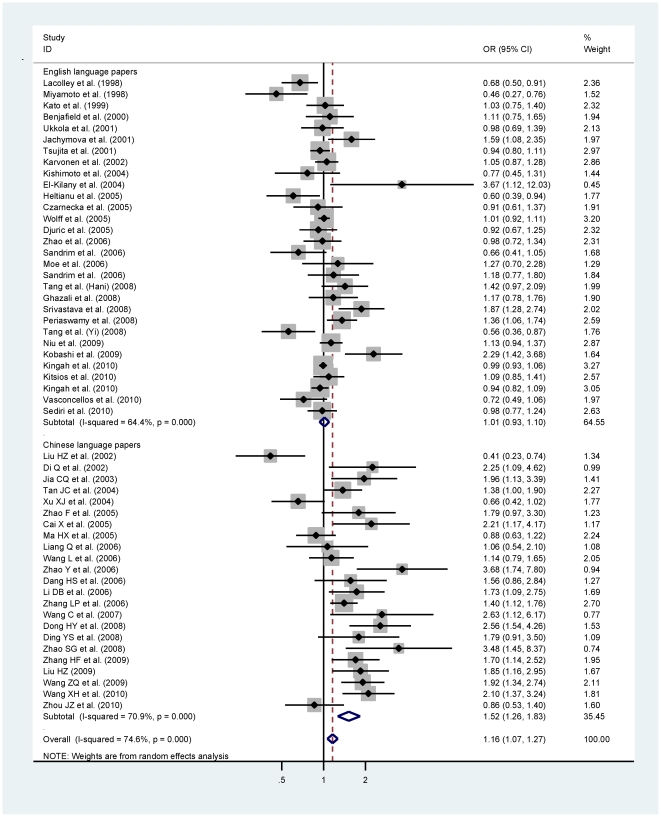
Allele comparison of eNOS G894T polymorphism for hypertension by language stratification.

**Figure 3:**
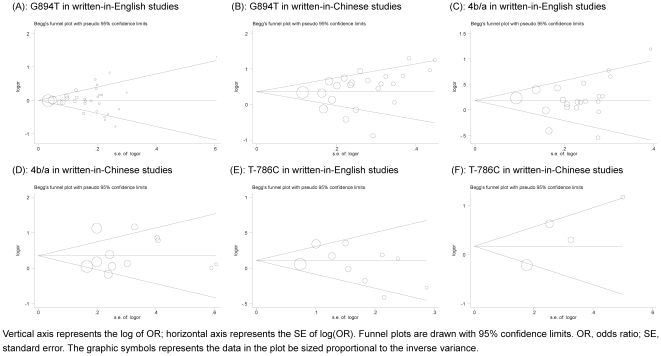
Funnel plots of allele comparisons for eNOS three polymorphisms upon stratification by published language. Vertical axis represents the log of OR; horizontal axis represents the SE of log(OR). Funnel plots are drawn with 95% confidence limits. OR, odds ratio; SE, standard error. The graphic symbols represents the data in the plot be sized proportional to the inverse variance.

Overall comparison of allele 894T with 894G, including both WIE and WIC studies, yielded an increased hypertension risk (OR = 1.16; 95% CI: 1.07–1.27; P = 0.001), even after Bonferroni correction, yet with strong evidence of heterogeneity (*I* ^2^ = 74.6%, P<0.0005) and high probability of publication bias as reflected by asymmetric funnel plot and significant Egger's test (P = 0.006). The magnitude of risk estimates for G894T genotype comparisons, as well as under dominant and recessive models were very similar to that in allele comparison (Table 1). Analyses by published language revealed that, whereas in WIC studies, the 894T may be associated with an increased hypertension risk even after Bonferroni correction (OR = 1.52; 95% CI: 1.26–1.83; P<0.0005), no clear evidence for a role of this variant was observed in WIE studies (Figure 2). Despite the significance of heterogeneity, publication bias was greatly improved in view of funnel plots (Figure 3A and 3B) and statistical tests (P_WIE_ = 0.702 and P_WIC_ = 0.171) in each subgroup. Irrespective of published languages, we observed significant association of 894T with hypertension among populations of Asian descent (OR = 1.32; 95% CI: 1.16–1.52; P<0.0005) and among populations of Chinese descent (OR = 1.40; 95% CI: 1.19–1.65; P<0.0005) (Figure S1).

**Table 1:** Comparisons of three studied *eNOS* polymorphisms in allele, genotype dominant and recessive models for hypertension risk.

Models	OR; 95% CI; P; *I* ^2^		
	G894T	4b/a	T-786C
All studies			
m vs. M	1.16; 1.07–1.27; 0.001; 74.6%*	1.29; 1.13–1.46; <0.0005; 60.6%*	1.12; 0.97–1.29; 0.137; 59.4%*
mM vs. MM	1.11; 1.00–1.22; 0.044; 67.1%*	1.32; 1.16–1.51; <0.0005; 51.7%*	1.11; 0.94–1.30; 0.224; 52.3%*
mm vs. MM	1.18; 1.01–1.39; 0.041; 37.1%*	1.34; 1.01–1.79; 0.045; 11.8%	1.07; 0.82–1.40; 0.610; 21.3%
mM plus mm vs. MM	1.15; 1.04–1.27; 0.005; 71.5%*	1.33; 1.16–1.52; <0.0005; 57.3%*	1.14; 0.98–1.36; 0.142; 58.0%*
mm vs. mM plus MM	1.20; 1.04–1.38; 0.015; 30.3%*	1.28; 0.98–1.67; 0.069; 3.3%	1.09; 0.87–1.36; 0.457; 6.3%
Written-in-English Studies			
m vs. M	1.01; 0.93–1.10; 0.748; 64.4%*	1.21; 1.06–1.96; 0.005; 52.8%*	1.08; 0.94–1.25; 0.255; 54.2%*
mM vs. MM	0.98; 0.88–1.08; 0.627; 61.7%	1.19; 1.05–1.36; 0.009; 35.2%*	1.09; 0.94–1.25; 0.247; 32.7%
mm vs. MM	1.03; 0.88–1.19; 0.742; 21.4%	1.44; 1.05–1.40; 0.006; 46.4%*	1.08; 0.77–1.52; 0.656; 40.3%*
mM plus mm vs. MM	1.00; 0.90–1.10; 0.932; 63.5%*	1.22; 1.05–1.40; 0.006; 46.4%*	1.10; 0.94–1.30; 0.235; 46.3%*
mm vs. mM plus MM	1.05; 0.95–1.15; 0.345; 0.0%	1.38; 1.03–1.86; 0.032; 0.0%	1.07; 0.80–1.43; 0.656; 31.7%
Written-in-Chinese Studies			
m vs. M	1.52; 1.26–1.83; <0.0005; 70.9%*	1.47; 1.11–1.96; 0.008; 68.8%*	1.46; 0.83–2.58; 0.189; 75.4%*
mM vs. MM	1.43; 1.17–1.74; <0.0005; 60.2%*	1.67; 1.25–2.22; <0.0005; 59.7%*	1.61; 0.74–3.48; 0.226; 79.2%*
mm vs. MM	1.94; 1.35–2.77; <0.0005; 28.4%	1.17; 0.60–2.28; 0.649; 25.8%	0.92; 0.52–1.62; 0.777; 0.0%
mM plus mm vs. MM	1.53; 1.25–1.88; <0.0005; 66.1%*	1.60; 1.19–2.16; 0.002; 65.7%*	1.56; 0.78–3.14; 0.207; 78.3%*
mm vs. mM plus MM	1.78; 1.30–2.44; <0.0005; 26.4%	1.06; 0.57–1.98; 0.853; 18.6%	1.01; 0.58–1.75; 0.968; 0.0%

*Abbreviations:* M, major or wild allele; m, minor or mutant allele; OR, odds ratio; 95% CI, 95% confidence interval.

For G894T, 4b/a and T-786C polymorphisms, the mutant alleles were 894T, 4a and -786C, respectively.

* P<0.1 for between-study heterogeneity test.

In ethnicity-stratified analyses, no significance was reached in allele model for WIE studies among Asians (OR = 1.09; P = 0.353), Arabs (OR = 1.66; P = 0.435), Whites (OR = 0.99; P = 0.828) and Blacks (OR = 0.85; P = 0.311), whereas contrastingly, the odds of developing hypertension were remarkably potentiated, albeit suffering heterogeneity, in WIC studies, except for a Kazakh study (Figure S2).

Additional stratification by study design showed slightly increased risk in hospital-based studies compared with population-based studies for both WIE and WIC studies, with significance reached in only WIC studies (Figure S3). After excluding one study with gestational hypertension, no material changes were observed from the above estimates.

To investigate the possible sources of heterogeneity, we meta-regressed the genetic effects of G894T polymorphism on explanatory variables including averaged levels of age, gender (male percent), study design, BMI, TG, TC, and HDLC. Unfortunately, the confounders under study showed no statistical contribution to the prediction of hypertension in allele/genotype models for both WIE and WIC studies (data not shown).

### 4b/a and Hypertension

**Figure 4:**
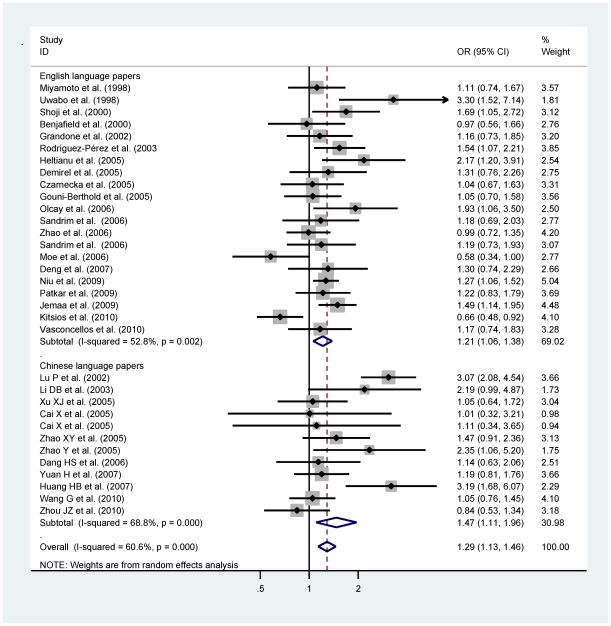
Allele comparison of eNOS 4b/a polymorphism for hypertension by language stratification.

Considering the funnel plots (Figure 3C and 3D) and statistical tests, comparisons of allele (P_Egger_ = 0.289), genotype (4a/4b versus 4a/4a: P_Egger_ = 0.145; 4a/4a versus 4a/4a: P_Egger_ = 0.849), dominant (P_Egger_ = 0.213) and recessive (P_Egger_ = 0.801) models in all qualified publications yielded low probability of publication bias. The significant overall estimate of allele 4a versus 4b was 1.29 (95% CI: 1.13–1.46; P<0.0005) for all studies, 1.21 (95% CI: 1.06–1.38; P = 0.005) for WIE studies and 1.47 (95% CI: 1.11–1.96; P = 0.008) for WIC studies, whereas there was strong evidence of between-study heterogeneity (P<0.005) (Figure 4). Comparisons of genotype 4a4b with 4b4b and in dominant model had the similar magnitude with allele model for all, WIE and WIC studies, whereas the homogeneous genotype and recessive comparisons were strengthened in only WIE studies (Table 1). Still, heterogeneity tingled these comparisons (P<0.01). When analyses were restricted to WIC studies, the significance persisted for each comparisons, and there was no publication bias, but significant between-study heterogeneity (Table 1).

Subgroup analyses were conducted to seek explanations for heterogeneity. Considering the study power, only those subgroups with ≥3 independent populations were reported. Comparison of allele 4a with 4b was significant, even after Bonferroni correction, in Asians (OR = 1.42; 95% CI: 1.16–1.72; P<0.0005), but not in Whites (OR = 1.14; 95% CI: 0.91–1.44; P = 0.261) (Figure S4). Further stratification by study design consistently presented significant associations in both population-based studies (OR = 1.25; 95% CI: 1.02–1.53; P = 0.032) and hospital-based studies (OR = 1.30; 95% CI: 1.11–1.52; P = 0.001) (Figure S5). When applying Bonferroni correction, only in hospital-based studies was there significant. In addition, after stratification upon disease endpoint, 4b/a polymorphism was not significantly associated with gestational hypertension across all genetic models, and significance persisted for essential hypertension (Figure S6). Furthermore in all studies, meta-regression of 4b/a polymorphism on averaged levels of age, gender (male percent), study design, BMI, TG, TC, and HDLC failed to identify any significance, even by published language stratification (data not shown).

### T−786C and Hypertension

**Figure 5:**
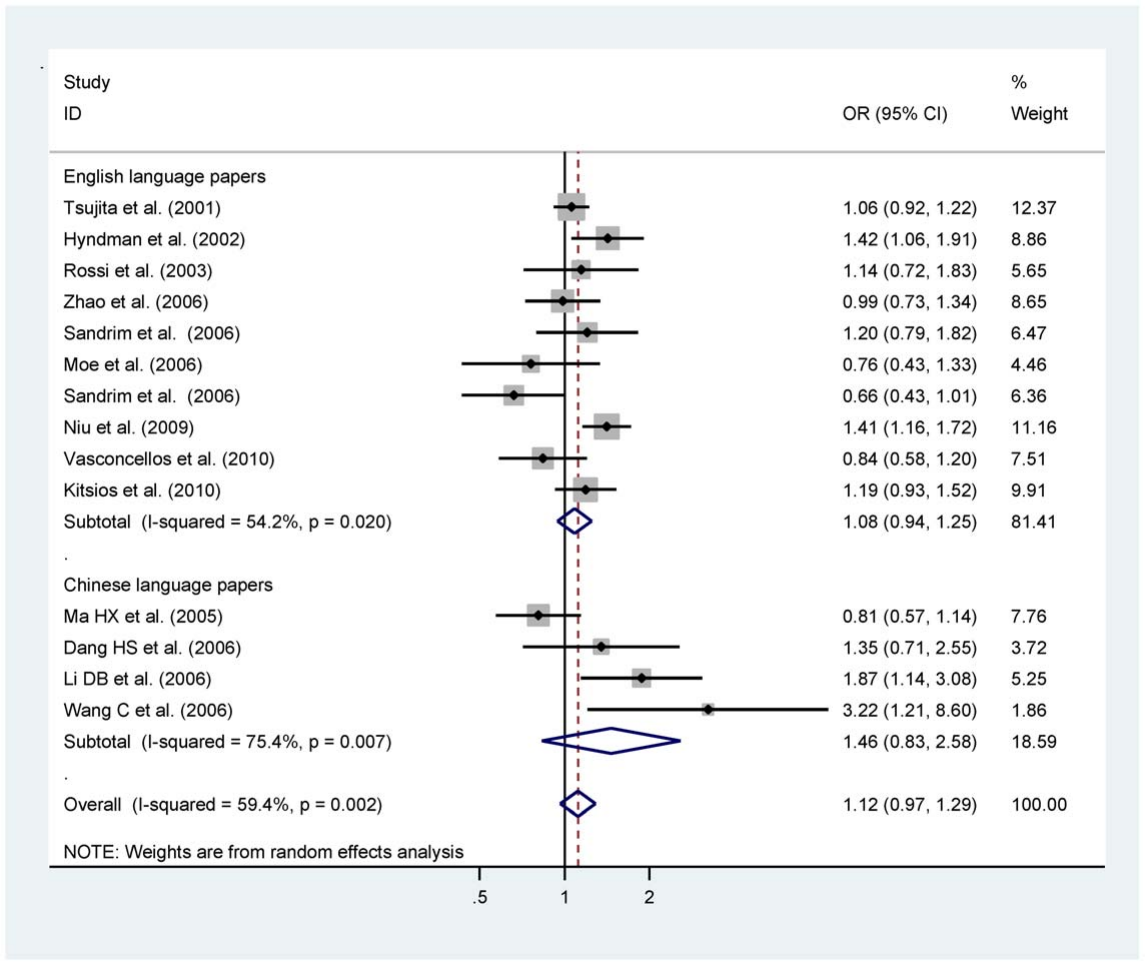
Allele comparison of eNOS T−786C polymorphism for hypertension by language stratification.

Besides the suggestive symmetry of funnel plots (Figure 3E and 3F), Egger's test indicated no publication bias in all studies for allele (P = 0.889), genotype (−786TC versus −786TT: P = 0.737; −786CC versus −786TT: P = 0.636), dominant (P = 0.996) and recessive (P = 0.454) comparisons. Overall comparison of allele −786C with −786T showed null association of this polymorphism with hypertension (OR = 1.12; 95% CI: 0.97–1.29; P = 0.137), even in subgroups by language (Figure 5). Similar associations were identified in genotype, dominant and recessive models (Table 1). There was no potential heterogeneity for the homogenous genotype and recessive comparisons (P>0.1). Results in Chinese populations still showed null associations for all models, and suffered significant heterogeneity except for homogeneous genotype and recessive comparisons, and no publication bias was observed.

Likewise, stratification by study design showed no significance for all studies, as well as for WIE and WIC studies (Figure S7). However, subgroup analyses by ethnicity suggested positivity, even after Bonferroni correction, for −786C allele (OR = 1.25; 95% CI: 1.06–1.47; P = 0.007) and −786CC genotype (OR = 1.69; 95% CI: 1.20–2.38; P = 0.003) with increased hypertension risk in Whites, even for the dominant and recessive models. Additionally, we meta-regressed T−786C polymorphism on averaged levels of age, gender (male percent), study design, BMI, TG, TC, and HDLC, and found no significant associations at all (data not shown).

## Discussion

Although our overall association produced significance for *eNOS* G894T and 4ab polymorphisms in Asians and T−786C polymorphism in Whites, the indications of between-study heterogeneity and publication bias, albeit disturbing, should merit serious consideration. To the best of our knowledge, this is the largest meta-analysis so far to investigate the association of *eNOS* three common polymorphisms with hypertension from the English and Chinese-published literature.

To avoid the disturbance of publication bias, we divided studies into characteristic-homogeneous groups such as the published language, and this situation has been greatly improved. For example, association of G894T polymorphism with hypertension showed high probability of publication bias in overall analyses, whereas none was observed in published language-stratified subgroups. At this point, we interestingly found remarkable heterogeneous associations by showing null association of 894T allele with hypertension in WIE studies, which was consistent with the results of two previous meta-analyses [49], [50]. Contrastingly, in this study, 894T allele carriers had a 52% increased risk in WIC studies, and a 40% and 32% increased risk, while suffering significant heterogeneity and publication bias, in Chinese and Asian populations, respectively (Figure S1). However, this association exhibited no significance in Whites, suggesting the heterogeneous associations of G894T polymorphism in ethnicity-specific populations. In view of this divergence, it is highly suggested to construct a database of polymorphisms related to hypertension in each racial or ethnic group [51].

Although linkage information was lacking for *eNOS*, two previous powerful meta-analyses indicated positive signals between *eNOS* variation and hypertension by consistently predisposing 4b allele to an increased risk, although publication bias obscured each study [49], [50], which was in agreement with our overall estimates. Although the influence of publication bias was ‘artificially’ curbed in this study, existence of heterogeneity still toggled most comparisons. In ethnicity-stratified analysis, we found striking heterogeneity by showing 4a allele carriers were at increased hypertension risk in Asians, but not in Whites. Because 4b/a polymorphism is intronic, it is unlikely to be functional but might act as a marker in linkage disequilibrium with other functional polymorphisms in *eNOS* regulatory regions. On the other hand, 4b/a might interact with other polymorphisms to predisposing individuals to susceptibility or resistance to hypertension. As evidenced, Sandrim et al evaluated the *eNOS* haplotypes-based risk and demonstrated the haplotype −786C-4b-894G was linked to a protective effect on hypertension risk [52]. Likewise, our recent haplotype analyses also supported the potential interaction between polymorphisms 4b/a and T−786C [42]. However, whether this interaction affects production or bioavailability of eNOS remains an open question.

As indicated by the results of previous meta-analyses [49], [50] and the present study, the *eNOS* T−786C polymorphism might not be a predisposing marker for hypertension. However, we extended this finding by showing significant association of T−786C in Whites. Factually, experimental studies have confirmed a functional role of this polymorphism on *eNOS* transcription activity by showing that the −786C-4b combination had the highest transcriptional activity [53]. Herein, because the single-locus-based nature of meta-analysis precluded the possibility of gene-gene and gene-environment interactions, as well as haplotype-based effects in this study, it is highly suggested that additional studies assessing these aspects will be necessary.

To seek explanations for heterogeneity, besides subgroup analyses, an alternative approach is to perform a multivariate meta-analysis, in the form of a meta-regression, with the inclusion of covariates within this framework. This approach enables the moderating effect of a covariate, such as age or gender, to be tested formally. Unfortunately in this study, performing random-effects meta-regression analyses on various study-level covariates failed to provide any significant findings for all polymorphisms under study. However, it is important to bear in mind that meta-regression, although enabling covariates to be considered, does not have the methodological rigor of a properly designed study that is intended to test the effect of these covariates formally [54]. Admittedly, one limitation facing this study was the number of studies that are available for inclusion. In fact, most studies did not report the study-level covariates of interest, precluding a more robust assessment of sources of heterogeneity.

Despite the clear strengths of our study including large sample sizes and comprehensive evaluation of *eNOS* variation, some limitations merit serious consideration. First, all included studies had the cross-sectional design, which precludes further comments on cause-effect relationship [55]. Second, for hypertension association studies, most studies have recruited subjects aged ≥50 years, for whom environmental factors are likely to contribute more prominently than the genetic component to the development of hypertension, suggesting that large association studies in a younger hypertensive subjects are of added interest. Third, we cannot retrieve common information from all these original publications upon some important intermediate phenotypes such as dietary salt intake in meta-regression models. Last but not least, in this study, we only focused on *eNOS* polymorphisms, and did not evaluate other genes or polymorphisms. It is possible that the potential roles of G894T or 4b/a or T−786C polymorphisms are diluted or masked by other gene-gene or gene-environment interactions. Thus, the jury must refrain from drawing a conclusion until a large, well-performed Chinese study confirms or refuses our results.

Taken together, via a comprehensive meta-analysis, we once again ascertained the role of *eNOS* G894T and 4b/a polymorphisms on the development of hypertension for Asian populations and T−786C polymorphism for Whites. Although the publication bias was maximally avoided, presence of between-study heterogeneity could not be fully explained by our subgroup and meta-regression analyses. Although further analyses are warranted to investigate *eNOS* adjacent markers in a wider context, future studies should center on gene-gene and gene-environment interactions, as well as haplotype patterns.

## Supporting Information

Figure S1 **Allele comparison of eNOS G894T polymorphism for hypertension in Chinese (left pane) and Asians (right pane).**
(TIFF)

Figure S2 **Ethnicity-stratified allele comparison of eNOS G894T polymorphism for hypertension in WIE (left pane) and WIC (right pane) studies.**
(TIFF)

Figure S3 **Study-design-stratified allele comparison of eNOS G894T polymorphism for hypertension in WIE (left pane) and WIC (right pane) studies.**
(TIFF)

Figure S4 **Ethnicity-stratified comparison of eNOS 4b/a polymorphism for hypertension in all studies.**
(TIFF)

Figure S5 **Study-design-stratified allele comparison of eNOS 4b/a polymorphism for hypertension in all studies.**
(TIFF)

Figure S6 **Endpoint-stratified allele comparison of eNOS 4b/a polymorphism for hypertension in all studies.**
(TIFF)

Figure S7 **Study-design-stratified allele comparison of eNOS T−786C polymorphism for hypertension in all studies.**
(TIFF)

Table S1 **The baseline characteristics of all qualified studies in this meta-analysis.**
(XLS)
