# Supplementary figures and images for: An Updated Meta-Analysis of Endothelial Nitric Oxide Synthase Gene: Three Well-Characterized Polymorphisms with Hypertension
Source: PLoS One. 2011 Sep 2;6(9):e24266. doi: 10.1371/journal.pone.0024266 (PMC3166328; doi:10.1371/journal.pone.0024266)

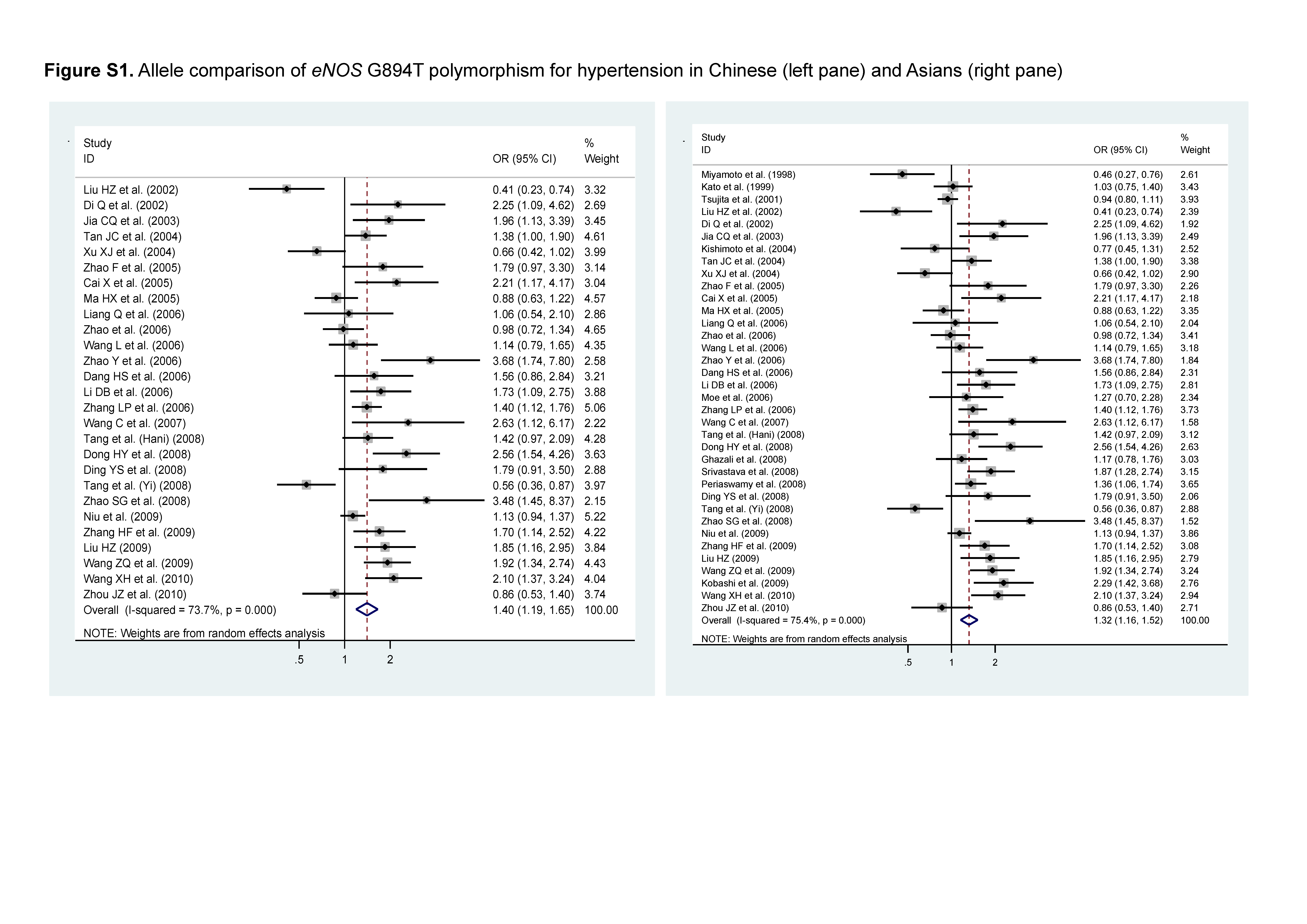

Supplement: Figure S1 — Allele comparison of eNOS G894T polymorphism for hypertension in Chinese (left pane) and Asians (right pane). (TIFF) [file pone.0024266.s001.tiff]

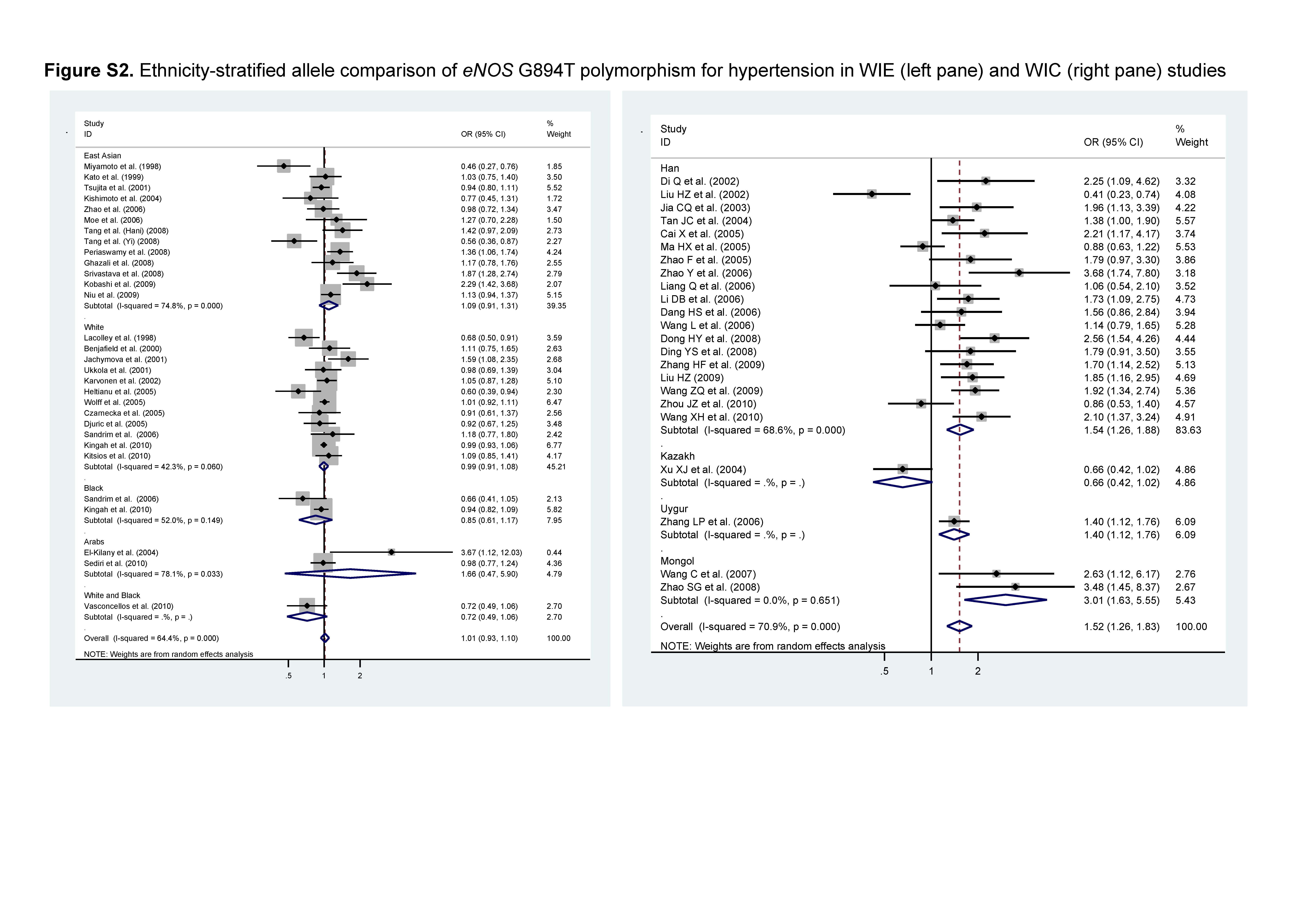

Supplement: Figure S2 — Ethnicity-stratified allele comparison of eNOS G894T polymorphism for hypertension in WIE (left pane) and WIC (right pane) studies. (TIFF) [file pone.0024266.s002.tiff]

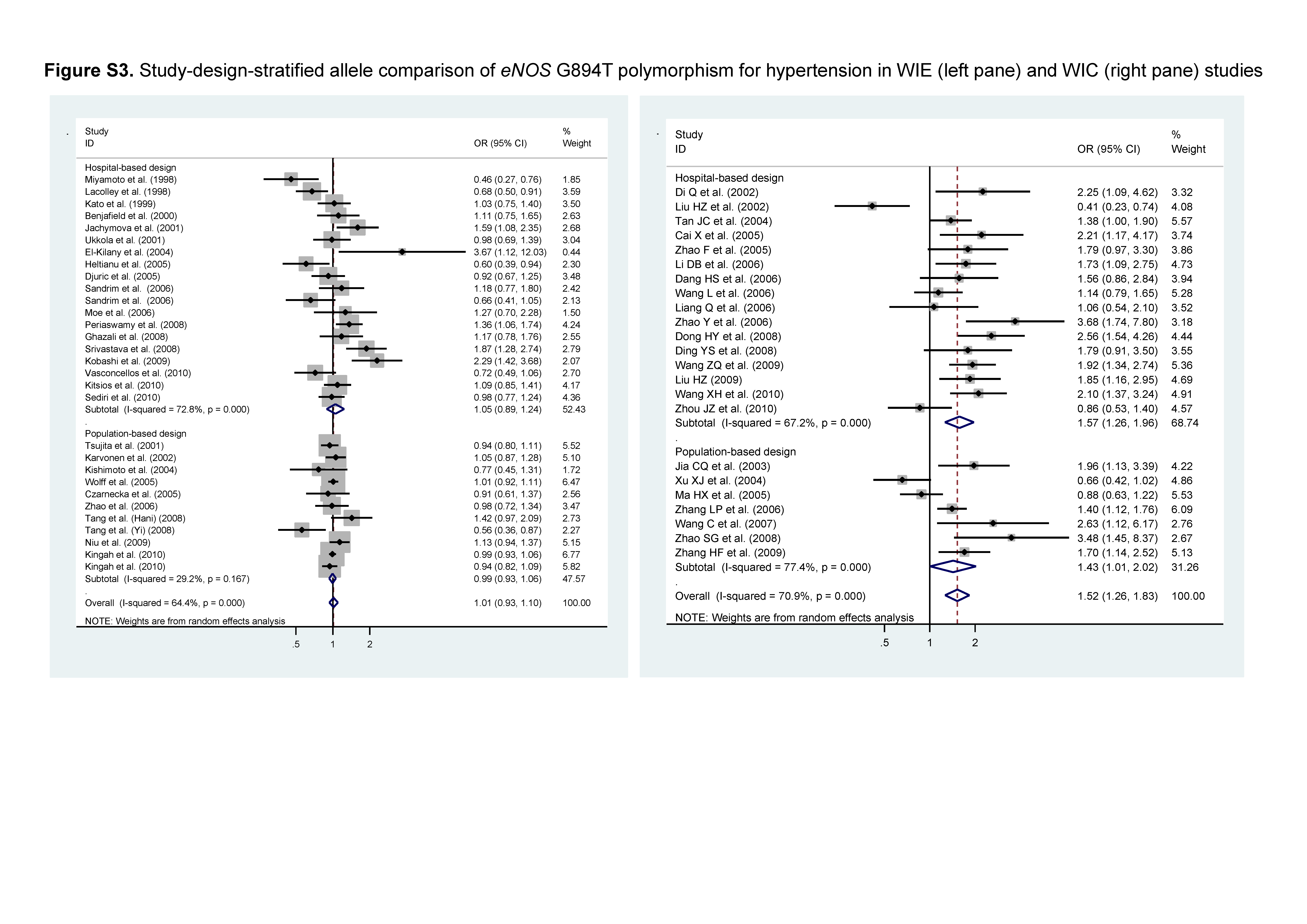

Supplement: Figure S3 — Study-design-stratified allele comparison of eNOS G894T polymorphism for hypertension in WIE (left pane) and WIC (right pane) studies. (TIFF) [file pone.0024266.s003.tiff]

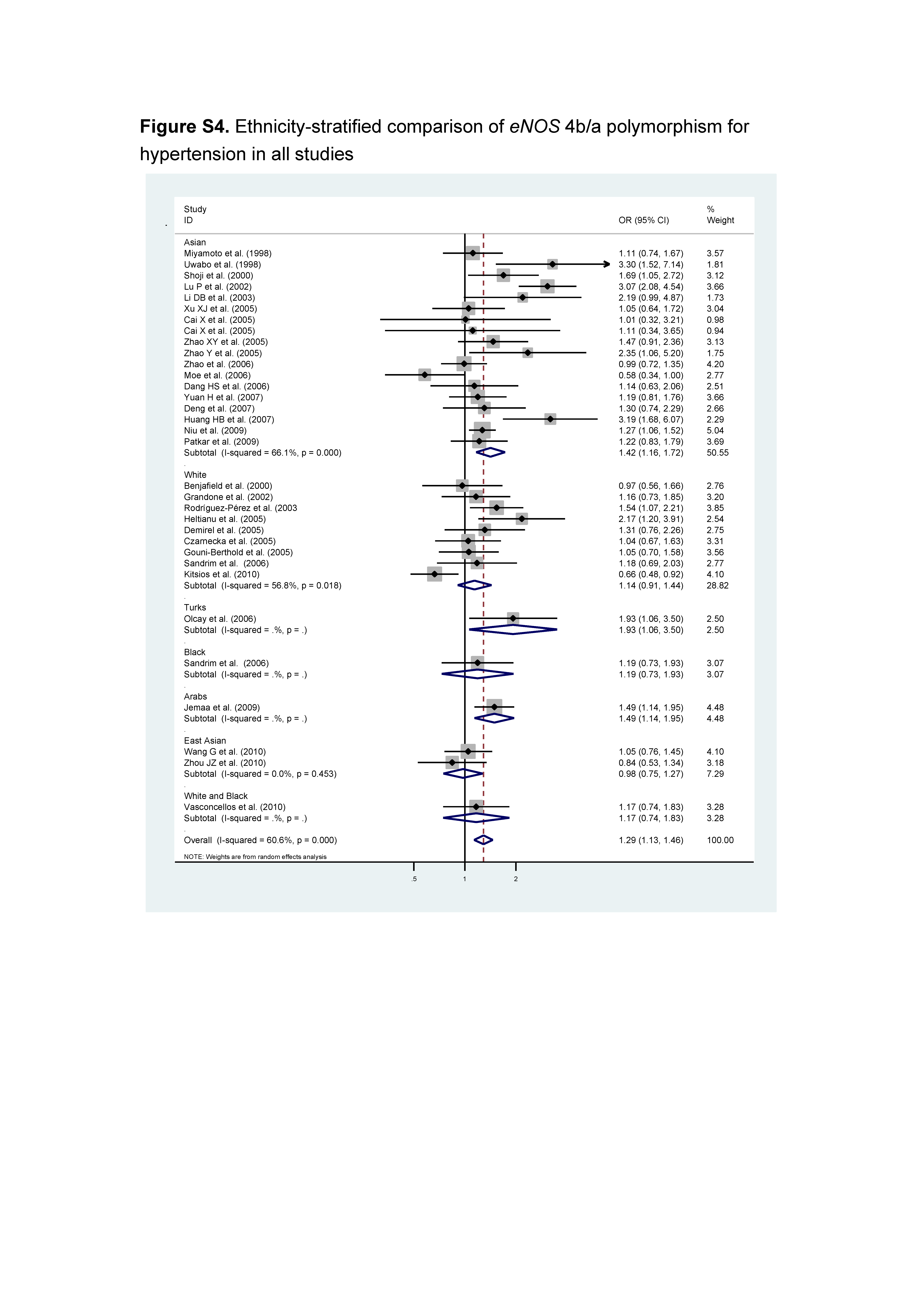

Supplement: Figure S4 — Ethnicity-stratified comparison of eNOS 4b/a polymorphism for hypertension in all studies. (TIFF) [file pone.0024266.s004.tiff]

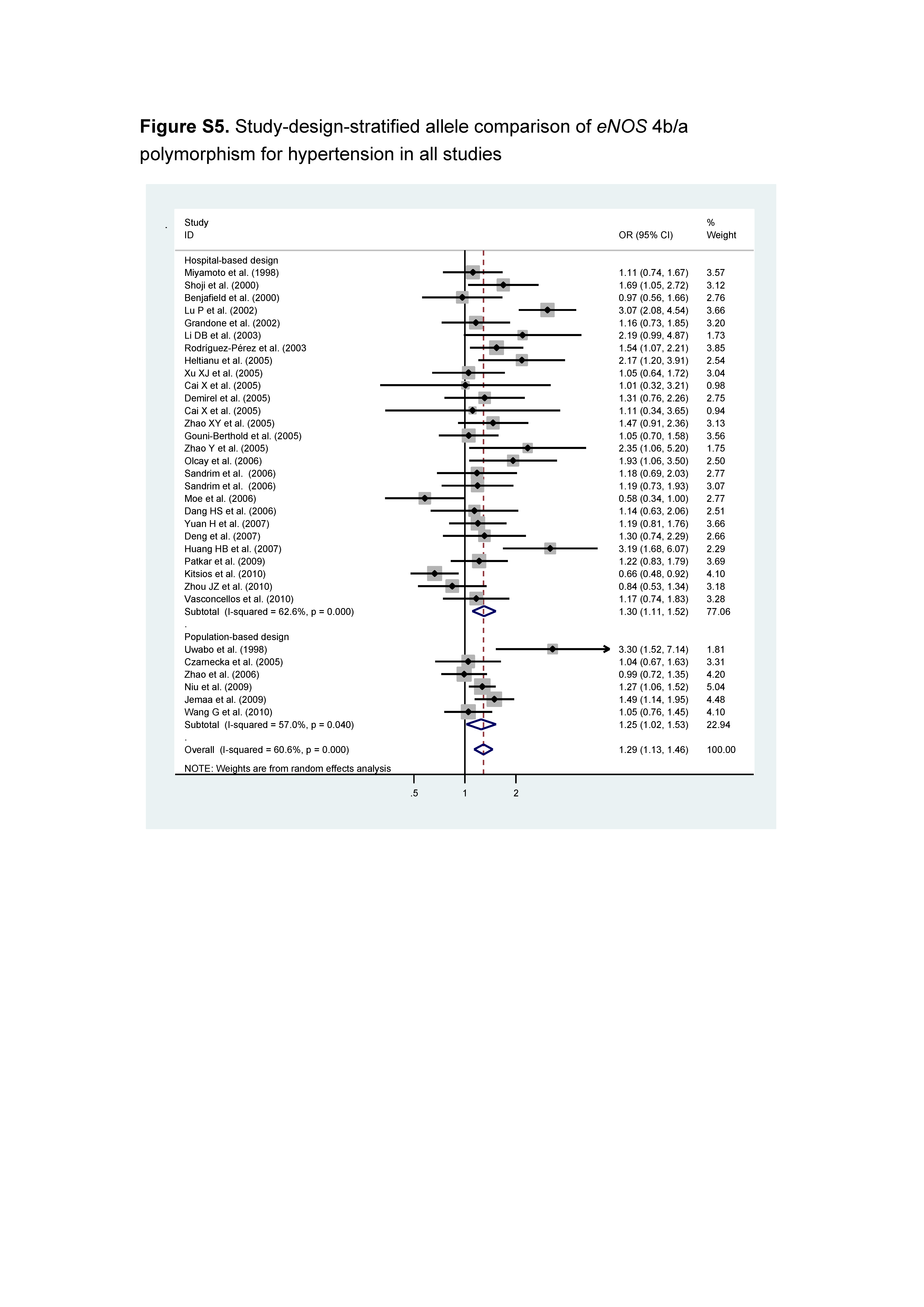

Supplement: Figure S5 — Study-design-stratified allele comparison of eNOS 4b/a polymorphism for hypertension in all studies. (TIFF) [file pone.0024266.s005.tiff]

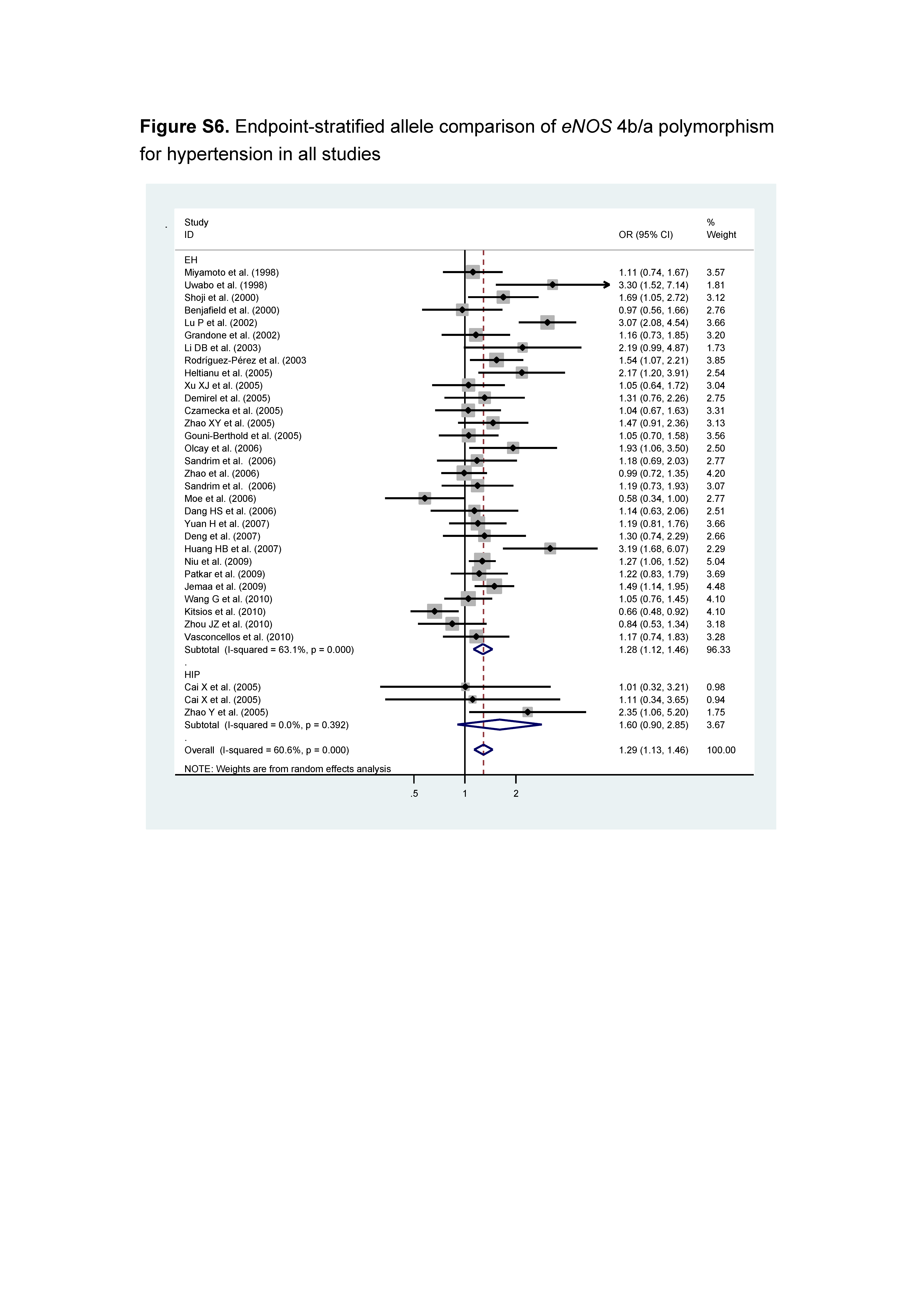

Supplement: Figure S6 — Endpoint-stratified allele comparison of eNOS 4b/a polymorphism for hypertension in all studies. (TIFF) [file pone.0024266.s006.tiff]

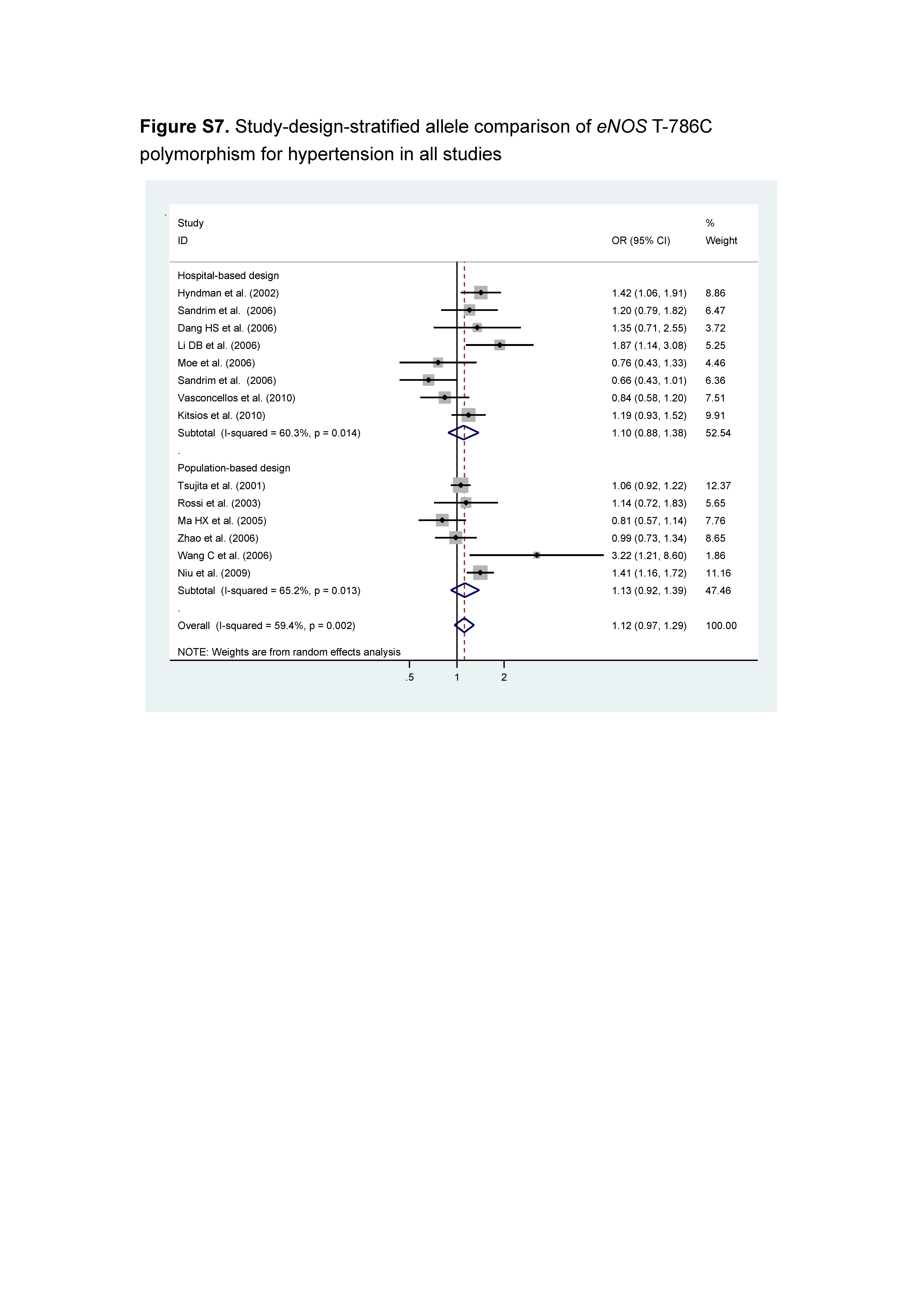

Supplement: Figure S7 — Study-design-stratified allele comparison of eNOS T−786C polymorphism for hypertension in all studies. (TIFF) [file pone.0024266.s007.tiff]
